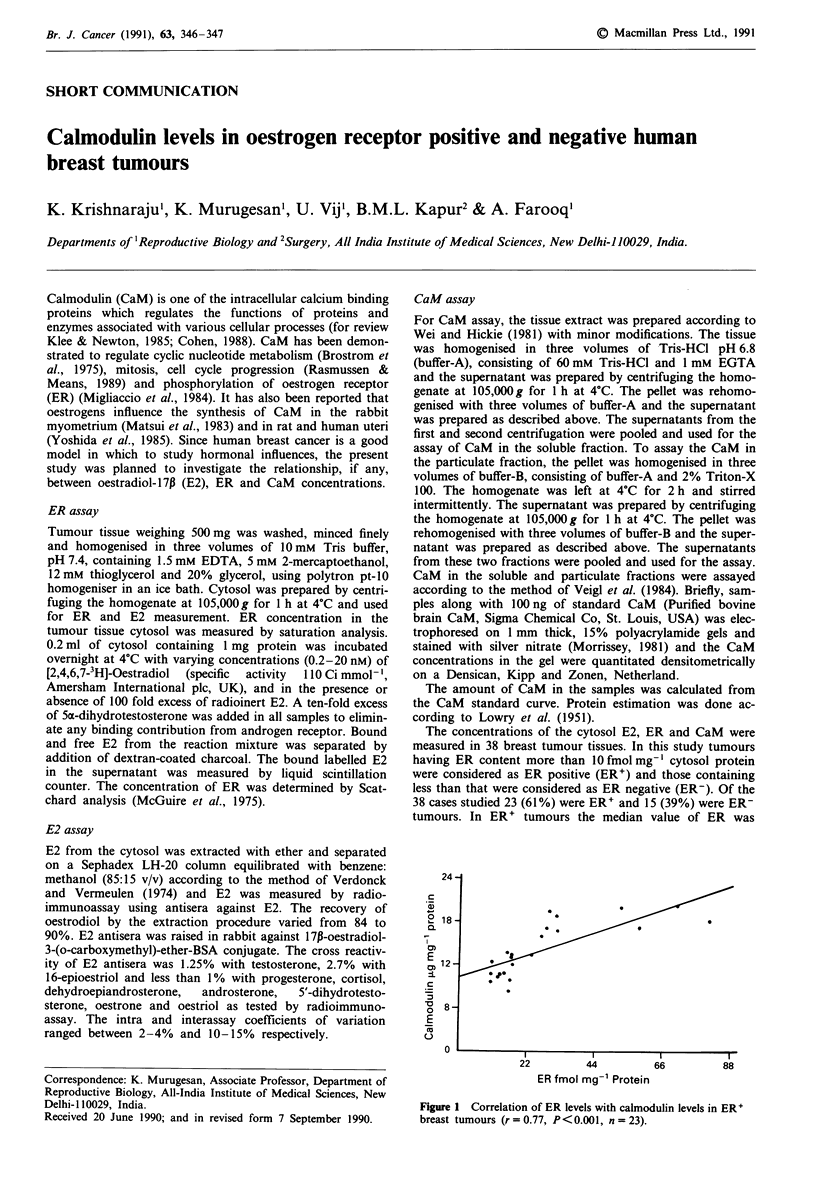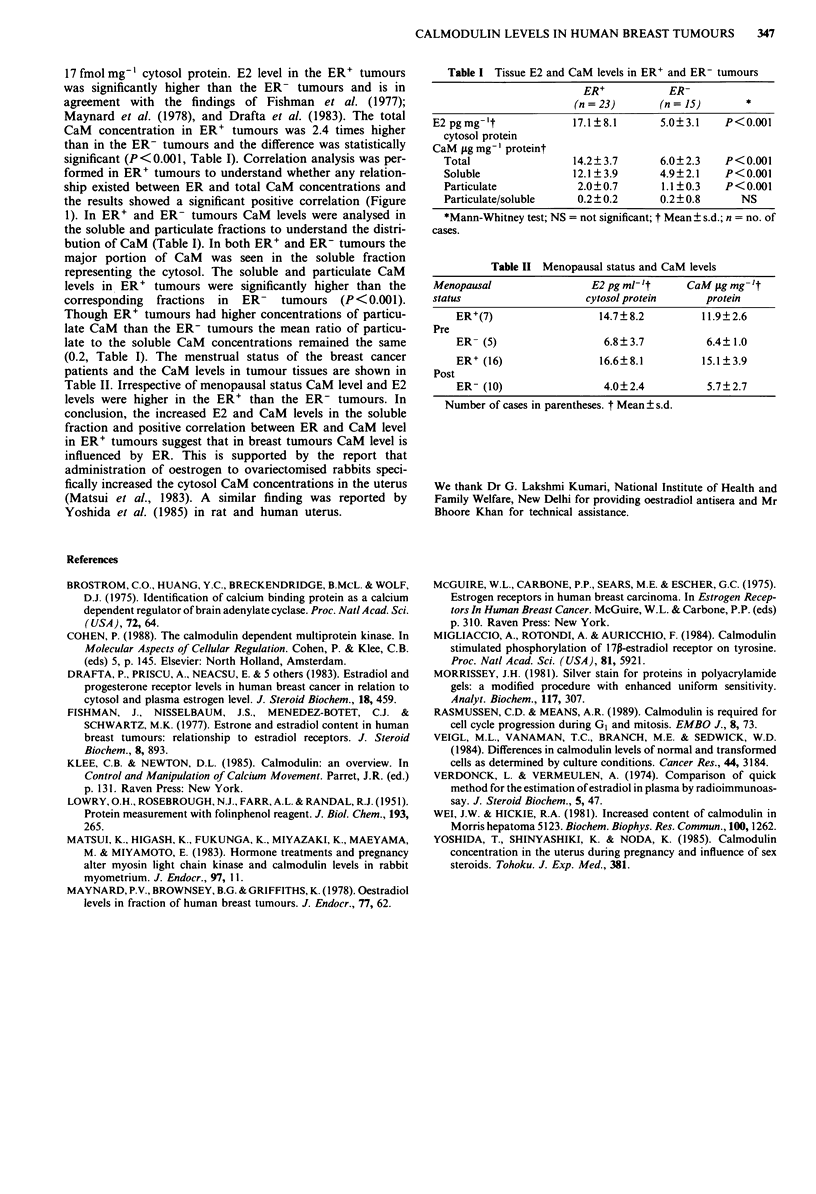# Calmodulin levels in oestrogen receptor positive and negative human breast tumours.

**DOI:** 10.1038/bjc.1991.82

**Published:** 1991-03

**Authors:** K. Krishnaraju, K. Murugesan, U. Vij, B. M. Kapur, A. Farooq

**Affiliations:** Department of Reproductive Biology, All India Institute of Medical Sciences, New Delhi.


Br. J. Cancer (1991), 63, 346-347 © Macmillan Press Ltd., 1991

SHORT COMMUNICATION

### Calmodulin levels in oestrogen receptor positive and negative human breast tumours

K. Krishnaraju[1], K. Murugesan[1], U. Vij[1], B.M.L. Kapur[2] & A. Farooq[1]

*Departments of [1]Reproductive Biology and [2]Surgery, All India Institute of Medical Sciences, New Delhi-110029, India.*

Calmodulin (CaM) is one of the intracellular calcium binding proteins which regulates the functions of proteins and enzymes associated with various cellular processes (for review Klee & Newton, 1985; Cohen, 1988). CaM has been demonstrated to regulate cyclic nucleotide metabolism (Brostrom *et al.*, 1975), mitosis, cell cycle progression (Rasmussen & Means, 1989) and phosphorylation of oestrogen receptor (ER) (Migliaccio *et al.*, 1984). It has also been reported that oestrogens influence the synthesis of CaM in the rabbit myometrium (Matsui *et al.*, 1983) and in rat and human uteri (Yoshida *et al.*, 1985). Since human breast cancer is a good model in which to study hormonal influences, the present study was planned to investigate the relationship, if any, between oestradiol-17β (E2), ER and CaM concentrations.

*ER assay*

Tumour tissue weighing 500 mg was washed, minced finely and homogenised in three volumes of 10 mM Tris buffer, pH 7.4, containing 1.5 mM EDTA, 5 mM 2-mercaptoethanol, 12 mM thioglycerol and 20% glycerol, using polytron pt-10 homogeniser in an ice bath. Cytosol was prepared by centrifuging the homogenate at 105,000 *g* for 1 h at 4°C and used for ER and E2 measurement. ER concentration in the tumour tissue cytosol was measured by saturation analysis. 0.2 ml of cytosol containing 1 mg protein was incubated overnight at 4°C with varying concentrations (0.2–20 nM) of [2,4,6,7-$^3$H]-Oestradiol (specific activity 110 Ci mmol$^{-1}$, Amersham International plc, UK), and in the presence or absence of 100 fold excess of radioinert E2. A ten-fold excess of 5α-dihydrotestosterone was added in all samples to eliminate any binding contribution from androgen receptor. Bound and free E2 from the reaction mixture was separated by addition of dextran-coated charcoal. The bound labelled E2 in the supernatant was measured by liquid scintillation counter. The concentration of ER was determined by Scatchard analysis (McGuire *et al.*, 1975).

*E2 assay*

E2 from the cytosol was extracted with ether and separated on a Sephadex LH-20 column equilibrated with benzene: methanol (85:15 v/v) according to the method of Verdonck and Vermeulen (1974) and E2 was measured by radioimmunoassay using antisera against E2. The recovery of oestrodiol by the extraction procedure varied from 84 to 90%. E2 antisera was raised in rabbit against 17β-oestradiol-3-(o-carboxymethyl)-ether-BSA conjugate. The cross reactivity of E2 antisera was 1.25% with testosterone, 2.7% with 16-epioestriol and less than 1% with progesterone, cortisol, dehydroepiandrosterone, androsterone, 5′-dihydrotestosterone, oestrone and oestriol as tested by radioimmunoassay. The intra and interassay coefficients of variation ranged between 2–4% and 10–15% respectively.

*CaM assay*

For CaM assay, the tissue extract was prepared according to Wei and Hickie (1981) with minor modifications. The tissue was homogenised in three volumes of Tris-HCl pH 6.8 (buffer-A), consisting of 60 mM Tris-HCl and 1 mM EGTA and the supernatant was prepared by centrifuging the homogenate at 105,000 *g* for 1 h at 4°C. The pellet was rehomogenised with three volumes of buffer-A and the supernatant was prepared as described above. The supernatants from the first and second centrifugation were pooled and used for the assay of CaM in the soluble fraction. To assay the CaM in the particulate fraction, the pellet was homogenised in three volumes of buffer-B, consisting of buffer-A and 2% Triton-X 100. The homogenate was left at 4°C for 2 h and stirred intermittently. The supernatant was prepared by centrifuging the homogenate at 105,000 *g* for 1 h at 4°C. The pellet was rehomogenised with three volumes of buffer-B and the supernatant was prepared as described above. The supernatants from these two fractions were pooled and used for the assay. CaM in the soluble and particulate fractions were assayed according to the method of Veigl *et al.* (1984). Briefly, samples along with 100 ng of standard CaM (Purified bovine brain CaM, Sigma Chemical Co, St. Louis, USA) was electrophoresed on 1 mm thick, 15% polyacrylamide gels and stained with silver nitrate (Morrissey, 1981) and the CaM concentrations in the gel were quantitated densitometrically on a Densican, Kipp and Zonen, Netherland.

The amount of CaM in the samples was calculated from the CaM standard curve. Protein estimation was done according to Lowry *et al.* (1951).

The concentrations of the cytosol E2, ER and CaM were measured in 38 breast tumour tissues. In this study tumours having ER content more than 10 fmol mg$^{-1}$ cytosol protein were considered as ER positive (ER$^+$) and those containing less than that were considered as ER negative (ER$^-$). Of the 38 cases studied 23 (61%) were ER$^+$ and 15 (39%) were ER$^-$ tumours. In ER$^+$ tumours the median value of ER was

**Figure 1** Correlation of ER levels with calmodulin levels in ER$^+$ breast tumours ($r = 0.77$, $P < 0.001$, $n = 23$).

Correspondence: K. Murugesan, Associate Professor, Department of Reproductive Biology, All-India Institute of Medical Sciences, New Delhi-110029, India.
Received 20 June 1990; and in revised form 7 September 1990.

17 fmol mg$^{-1}$ cytosol protein. E2 level in the ER$^+$ tumours was significantly higher than the ER$^-$ tumours and is in agreement with the findings of Fishman *et al.* (1977); Maynard *et al.* (1978), and Drafta *et al.* (1983). The total CaM concentration in ER$^+$ tumours was 2.4 times higher than in the ER$^-$ tumours and the difference was statistically significant ($P<0.001$, Table I). Correlation analysis was performed in ER$^+$ tumours to understand whether any relationship existed between ER and total CaM concentrations and the results showed a significant positive correlation (Figure 1). In ER$^+$ and ER$^-$ tumours CaM levels were analysed in the soluble and particulate fractions to understand the distribution of CaM (Table I). In both ER$^+$ and ER$^-$ tumours the major portion of CaM was seen in the soluble fraction representing the cytosol. The soluble and particulate CaM levels in ER$^+$ tumours were significantly higher than the corresponding fractions in ER$^-$ tumours ($P<0.001$). Though ER$^+$ tumours had higher concentrations of particulate CaM than the ER$^-$ tumours the mean ratio of particulate to the soluble CaM concentrations remained the same (0.2, Table I). The menstrual status of the breast cancer patients and the CaM levels in tumour tissues are shown in Table II. Irrespective of menopausal status CaM level and E2 levels were higher in the ER$^+$ than the ER$^-$ tumours. In conclusion, the increased E2 and CaM levels in the soluble fraction and positive correlation between ER and CaM level in ER$^+$ tumours suggest that in breast tumours CaM level is influenced by ER. This is supported by the report that administration of oestrogen to ovariectomised rabbits specifically increased the cytosol CaM concentrations in the uterus (Matsui *et al.*, 1983). A similar finding was reported by Yoshida *et al.* (1985) in rat and human uterus.

**Table I** Tissue E2 and CaM levels in ER$^+$ and ER$^-$ tumours

| | *ER$^+$* (*n* = 23) | *ER$^-$* (*n* = 15) | * |
|---|---|---|---|
| E2 pg mg$^{-1}$† cytosol protein | 17.1±8.1 | 5.0±3.1 | $P<0.001$ |
| CaM μg mg$^{-1}$ protein† | | | |
| Total | 14.2±3.7 | 6.0±2.3 | $P<0.001$ |
| Soluble | 12.1±3.9 | 4.9±2.1 | $P<0.001$ |
| Particulate | 2.0±0.7 | 1.1±0.3 | $P<0.001$ |
| Particulate/soluble | 0.2±0.2 | 0.2±0.8 | NS |

*Mann-Whitney test; NS = not significant; † Mean±s.d.; *n* = no. of cases.

**Table II** Menopausal status and CaM levels

| *Menopausal status* | | *E2 pg ml$^{-1}$† cytosol protein* | *CaM μg mg$^{-1}$† protein* |
|---|---|---|---|
| Pre | ER$^+$(7) | 14.7±8.2 | 11.9±2.6 |
| | ER$^-$ (5) | 6.8±3.7 | 6.4±1.0 |
| Post | ER$^+$ (16) | 16.6±8.1 | 15.1±3.9 |
| | ER$^-$ (10) | 4.0±2.4 | 5.7±2.7 |

Number of cases in parentheses. † Mean±s.d.

We thank Dr G. Lakshmi Kumari, National Institute of Health and Family Welfare, New Delhi for providing oestradiol antisera and Mr Bhoore Khan for technical assistance.

### References

BROSTROM, C.O., HUANG, Y.C., BRECKENDRIDGE, B.McL. & WOLF, D.J. (1975). Identification of calcium binding protein as a calcium dependent regulator of brain adenylate cyclase. *Proc. Natl Acad. Sci. (USA)*, **72**, 64.

COHEN, P. (1988). The calmodulin dependent multiprotein kinase. In *Molecular Aspects of Cellular Regulation*. Cohen, P. & Klee, C.B. (eds) 5, p. 145. Elsevier: North Holland, Amsterdam.

DRAFTA, P., PRISCU, A., NEACSU, E. & 5 others (1983). Estradiol and progesterone receptor levels in human breast cancer in relation to cytosol and plasma estrogen level. *J. Steroid Biochem.*, **18**, 459.

FISHMAN, J., NISSELBAUM, J.S., MENEDEZ-BOTET, C.J. & SCHWARTZ, M.K. (1977). Estrone and estradiol content in human breast tumours: relationship to estradiol receptors. *J. Steroid Biochem.*, **8**, 893.

KLEE, C.B. & NEWTON, D.L. (1985). Calmodulin: an overview. In *Control and Manipulation of Calcium Movement*. Parret, J.R. (ed.) p. 131. Raven Press: New York.

LOWRY, O.H., ROSEBROUGH, N.J., FARR, A.L. & RANDAL, R.J. (1951). Protein measurement with folinphenol reagent. *J. Biol. Chem.*, **193**, 265.

MATSUI, K., HIGASH, K., FUKUNGA, K., MIYAZAKI, K., MAEYAMA, M. & MIYAMOTO, E. (1983). Hormone treatments and pregnancy alter myosin light chain kinase and calmodulin levels in rabbit myometrium. *J. Endocr.*, **97**, 11.

MAYNARD, P.V., BROWNSEY, B.G. & GRIFFITHS, K. (1978). Oestradiol levels in fraction of human breast tumours. *J. Endocr.*, **77**, 62.

MCGUIRE, W.L., CARBONE, P.P., SEARS, M.E. & ESCHER, G.C. (1975). Estrogen receptors in human breast carcinoma. In *Estrogen Receptors In Human Breast Cancer*. McGuire, W.L. & Carbone, P.P. (eds) p. 310. Raven Press: New York.

MIGLIACCIO, A., ROTONDI, A. & AURICCHIO, F. (1984). Calmodulin stimulated phosphorylation of 17β-estradiol receptor on tyrosine. *Proc. Natl Acad. Sci. (USA)*, **81**, 5921.

MORRISSEY, J.H. (1981). Silver stain for proteins in polyacrylamide gels: a modified procedure with enhanced uniform sensitivity. *Analyt. Biochem.*, **117**, 307.

RASMUSSEN, C.D. & MEANS, A.R. (1989). Calmodulin is required for cell cycle progression during $G_1$ and mitosis. *EMBO J.*, **8**, 73.

VEIGL, M.L., VANAMAN, T.C., BRANCH, M.E. & SEDWICK, W.D. (1984). Differences in calmodulin levels of normal and transformed cells as determined by culture conditions. *Cancer Res.*, **44**, 3184.

VERDONCK, L. & VERMEULEN, A. (1974). Comparison of quick method for the estimation of estradiol in plasma by radioimmunoassay. *J. Steroid Biochem.*, **5**, 47.

WEI, J.W. & HICKIE, R.A. (1981). Increased content of calmodulin in Morris hepatoma 5123. *Biochem. Biophys. Res. Commun.*, **100**, 1262.

YOSHIDA, T., SHINYASHIKI, K. & NODA, K. (1985). Calmodulin concentration in the uterus during pregnancy and influence of sex steroids. *Tohoku. J. Exp. Med.*, **381**.